# Palmar-divergent dislocation of the scaphoid and the lunate

**DOI:** 10.1007/s10195-011-0131-5

**Published:** 2011-02-22

**Authors:** Shingo Komura, Tatsuo Yokoi, Yasushi Suzuki

**Affiliations:** 1 Department of Orthopaedic Surgery, Gifu Prefectural General Medical Center, Gifu, 500-8717 Japan; 2 Department of Orthopaedic Surgery, Gifu Prefectural Gero Hot Spring Hospital, Gifu, Japan

**Keywords:** Dislocation, Scaphoid, Lunate, Surgical repair, Carpal interosseous ligament

## Abstract

We describe a patient with palmar-divergent dislocation of the scaphoid and lunate. After successful closed reduction, the scapholunate and lunotriquetral ligaments were sutured through the dorsal approach, and the anterior capsule was sutured through the palmar approach. The scapholunate and lunotriquetral joints were fixed with Kirschner wires for 7 weeks. At the 1-year follow-up, magnetic resonance imaging showed no evidence of avascular necrosis of the scaphoid or lunate, and radiographs showed no evidence of the dorsal and volar intercalated segment instability patterns associated with carpal instability. However, flexion of the scaphoid and a break in Gilula’s line remained. To our knowledge, this is the first report showing treatment of palmar-divergent dislocation of the scaphoid and lunate by suturing the carpal interosseous ligaments.

## Introduction

Simultaneous palmar dislocation of the scaphoid and lunate is rare [1–7] and has been classified into two types depending on whether or not the scapholunate ligament is intact. Ten patients with dislocation of the scaphoid and lunate as a unit have been described to date, as well as six patients with divergent dislocation [1–6]. The patient described here is therefore the seventh with palmar-divergent dislocation of the scaphoid and lunate. In this patient, the scapholunate and lunotriquetral ligaments were sutured through the dorsal approach, the anterior capsule was sutured through the palmar approach, and the scapholunate and lunotriquetral joints were fixed with Kirschner wires. To our knowledge, this is the first report in which interosseous ligaments were sutured by open surgery for divergent dislocation of the scaphoid and lunate.

## Case report

**Fig. 1:**
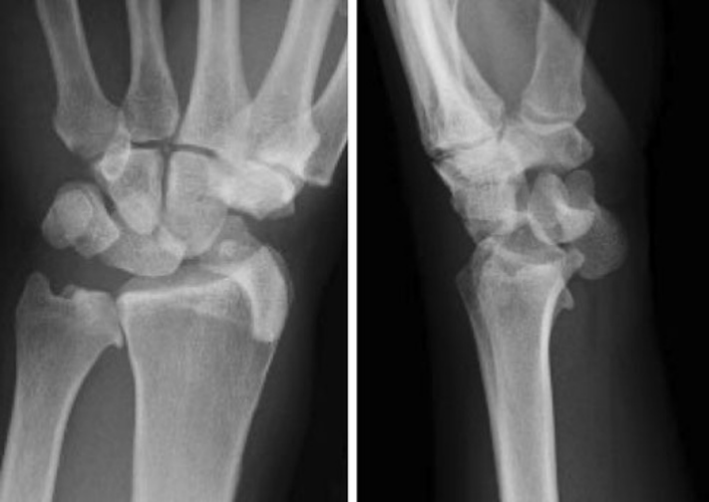
Radiographs at initial diagnosis showing palmar-divergent dislocation of the scaphoid and lunate

**Fig. 2:**
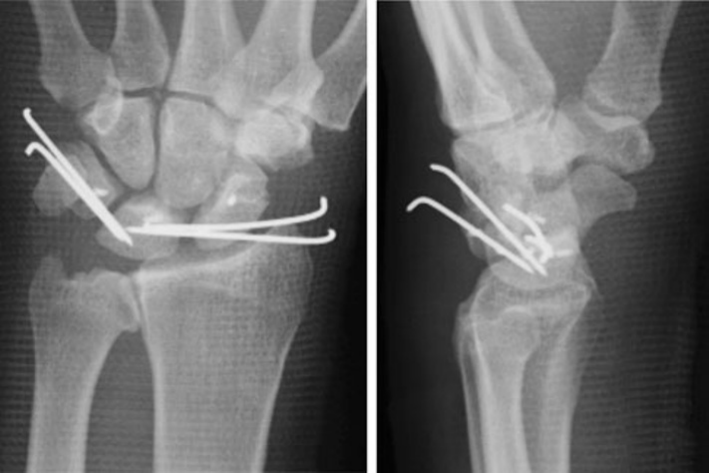
Postoperative radiographs showing good alignment of the carpal bones. The scapholunate angle was 54° and the radiolunate angle 6°. Gilula’s line was well-regulated

**Fig. 3:**
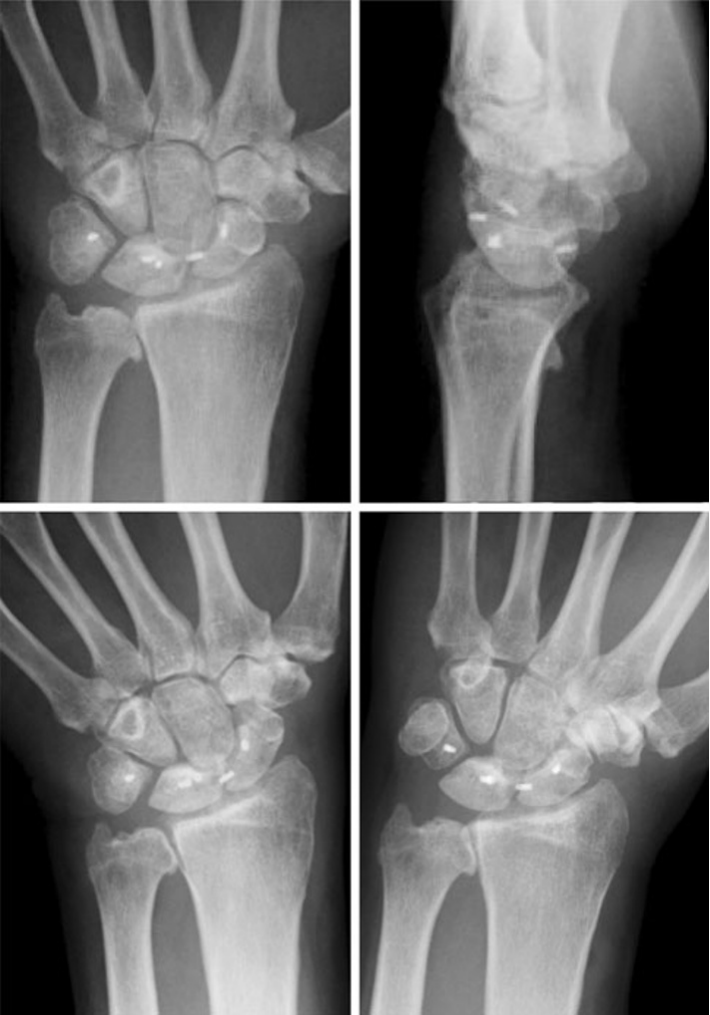
Radiographs at the 1-year follow-up. The scapholunate angle was 67° and the radiolunate angle 0°. Dorsal intercalated segment instability (DISI) deformity was not observed, although there was flexion of the scaphoid and a break in arc II of Gilula’s line at neutral and ulnar deviation

**Fig. 4:**
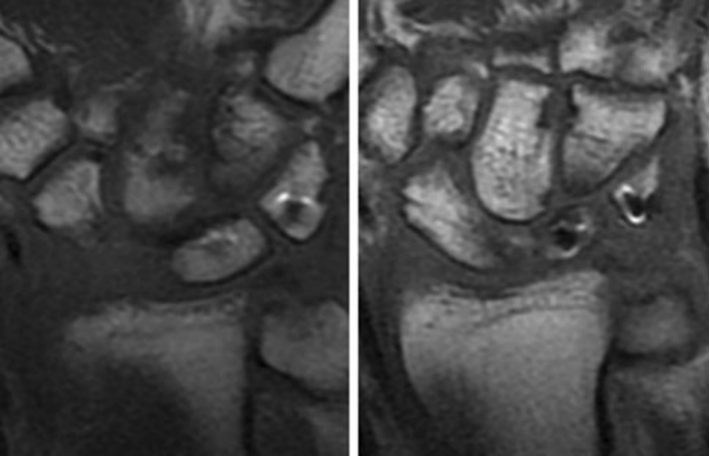
Magnetic resonance imaging at the 1-year follow-up showing no evidence of avascular necrosis of the scaphoid and lunate

A 46-year-old man who fell from a height of 1.5 m onto his left hand was brought to the emergency center of our hospital and underwent a medical examination. Radiography of the wrist revealed palmar-divergent dislocation of the scaphoid and lunate (Fig. 1) but with no neurovascular disturbance in the hand. Two hours after the injury, we performed closed reduction under local anesthesia. Although closed reduction was successful, severe carpal instability was observed. Seven days after the injury, open surgery was performed through the palmar and dorsal approaches. The dorsal approach showed ruptures of the scapholunate and lunotriquetral ligaments, which were sutured with anchors. The palmar approach showed an oblique tear of the anterior capsule, which was sutured with absorbable threads. Finally, the scapholunate and lunotriquetral joints were fixed with two Kirschner wires, inserted from the scaphoid to the lunate and from the triquetrum to the lunate, respectively, and the wires were buried under the skin (Fig. 2). A short arm plaster splint was applied postoperatively; 2 weeks later, it was changed to a removable splint and rehabilitation was started. As Kirschner wires remained in the carpal bones, range of motion (ROM) exercises of the wrist were restricted to avoid wire failure. At 7 weeks, the Kirschner wires and splint were removed, and the patient was started on intensive rehabilitation for an additional 3 months. At the 1-year follow-up, the patient had returned to normal life and work and had no pain in his wrist, although wrist motion was still restricted. Measurements of wrist and forearm ROM showed that right/left extension was 60/50°, flexion was 70/40°, supination was 90/80°, and pronation was 90/90°. A hand dynamometer showed that grip strength in his left hand was 16 kg compared with 27 kg on the contralateral (dominant) side. Although we observed no evidence of dorsal or volar intercalated segment instability pattern deformity, radiography showed a break in arc II of Gilula’s line between the lunate and triquetrum, as well as flexion deformity of the scaphoid (Fig. 3) [8]. Magnetic resonance imaging showed no evidence of avascular necrosis of the scaphoid and lunate (Fig. 4).

The patient provided written informed consent prior to inclusion in this study, which was authorized by the local ethics committee and performed in accordance with the ethical standards of the 1964 Declaration of Helsinki as revised in 2000.

## Discussion

As palmar-divergent dislocation of the scaphoid and lunate is rare, its optimal treatment remains unclear. In previous reports, two patients were treated by open reduction and cast immobilization [1, 2], two by open reduction and percutaneous pinning of the carpal bones and cast immobilization [3, 4], one by open reduction and suture of the anterior capsule and cast immobilization [5], and one by proximal row carpectomy (PRC) (Table 1). Carpal instability is severe in divergent dislocation due to ruptures of both the scapholunate and lunotriquetral ligaments. Therefore, it is difficult to stabilize the carpal bones and still retain sufficient wrist motion.

**Table 1:** Review of previous patients with divergent dislocation of the scaphoid and lunate

Author	Follow-up (months)	Surgical procedure	Approach	Immobilization (duration)	K-wire removal	Range of motion	Complications
Campbell [2]	12	Only open reduction	Palmar	Cast NR	–	Ext 1/2 Flex 1/3 of healthy side	None
Gordon [1]	12	Only open reduction	Palmar	Cast 4 weeks	–	Ext 15° Flex 25°	DISI
Kupfer [3]	42	Open reduction K-wire pinning (S-L)	Palmar & dorsal	Cast 4 months	4 months	Ext 25° Flex 0°	CRPS DISI AN (scaphoid, lunate)
Baulot [5]	42	Open reduction Anterior capsule suture	Palmar	Cast 6 weeks		Almost full	DISI
Kang [4]	18	Open reduction K-wire pinning (S-L/S-C)	Palmar	Cast 6 weeks	6 weeks	Almost full	None
Domeshek [6]	1	Proximal row carpectomy	Palmar & dorsal	Splint 1 month	–	NR	NR

*AN* avascular necrosis, *NR* not recorded, *K*-*wire* Kirschner wire, *S* scaphoid, *L* lunate, *T* triquetrum, *C* capitate, *DISI* dorsal intercalated segment instability, *CRPS* complex regional pain syndrome, *Ext* extension, *Flex* flexion

Among the methods recommended to repair, the anterior and posterior ligaments on both sides of the lunate are combined palmar and dorsal approaches [5], and open reduction and percutaneous pinning of the scapholunate and scaphocapitate joints without suture of the interosseous ligaments [4]. Although we found that suturing of the dorsal scapholunate and lunotriquetral ligaments provided a satisfactory outcome in our patient, wrist stiffness, carpal malalignment due to a break in arc II of Gilula’s line between the lunate and triquetrum, and flexion of the scaphoid still remained. Several problems arose during surgery and postoperative management. First, we should have sutured the palmar, not the dorsal, lunotriquetral ligament because the palmar ligament is stronger. This may have prevented the break in Gilula’s line. Moreover, in addition to fixing the scapholunate and lunotriquetral joints with Kirschner wires, we should have fixed the scaphocapitate joint to maximize anatomical carpal alignment. Fixation of the scaphocapitate joint may have prevented flexion deformity of the scaphoid. Thus, for reliable carpal stability, we recommend ligament repair and temporary joint fixation of the carpal bones. Subsequent wrist stiffness may be prevented by early removal of Kirschner wires after surgery and starting wrist exercises. Indeed, it may be possible to remove Kirschner wires earlier than 6 weeks when interosseous ligaments are sutured [4].

The injury to our patient may have been accompanied by avascular necrosis of the scaphoid and lunate [3]. PRC on a patient with a scapholunate dislocation and complete scaphoid extrusion resulted in a good clinical outcome [6], suggesting that PRC may eliminate avascular necrosis and avoid additional surgery in patients with this type of injury. However, although PRC has shown satisfactory clinical outcomes, postoperative ROM and grip strength averaged 50–70% and 60–90%, respectively, compared with the healthy side [9], outcomes similar to those observed in our patient. Therefore, except when unavoidable, we recommend surgical repair, especially for active young people and manual workers, with PRC considered a salvage procedure.

## References

[1] Gordon SL (1972). Scaphoid and lunate dislocation. Report of a case in a patient with peripheral neuropathy. J Bone Joint Surg Am.

[2] Campbell RD, Thompson TC, Lance EM, Adler JB (1965). Indications for open reduction of lunate and perilunate dislocations of the carpal bones. J Bone Joint Surg Am.

[3] Kupfer K (1986). Palmar dislocation of scaphoid and lunate as a unit: case report with special reference to carpal instability and treatment. J Hand Surg Am.

[4] Kang HJ, Shim DJ, Hahn SB, Kang ES (2003). Palmar divergent dislocation of scaphoid and lunate. Yonsei Med J.

[5] Baulot E, Perez A, Hallonet D, Grammont PM (1997). Scaphoid and lunate palmar divergent dislocation. Apropos of a case. Rev Chir Orthop Reparatrice Appar Mot.

[6] Domeshek LF, Harenberg PS, Rineer CA, Hadeed JG, Marcus JR, Erdmann D (2010). Total scapholunate dislocation with complete scaphoid extrusion: case report. J Hand Surg Am.

[7] Raemisch ME, Rotman MB (2004). Palmar dislocation of the scaphoid and lunate as a unit. Orthopedics.

[8] Gilula LA (1979). Carpal injuries: analytic approach and case exercises. AJR Am J Roentgenol.

[9] Edouard P, Vernay D, Martin S, Hirsch P, Bardoux S, Grange C, Claus D, Claise JM (2010). Proximal row carpectomy: is early postoperative mobilisation the right rehabilitation protocol?. Orthop Traumatol Surg Res.

